# How online public opinion evolves before and after policy adjustments in response to major public health emergencies

**DOI:** 10.3389/fpubh.2025.1438854

**Published:** 2025-06-09

**Authors:** Zhendong Niu, Yunyun Gao, Xusheng Wu, Qingyuan Hu, Dehua Hu

**Affiliations:** ^1^Department of Biomedical Informatics, School of Life Sciences, Central South University, Changsha, China; ^2^School of Information Management, Sun Yat-sen University, Guangzhou, China; ^3^Shenzhen Health Development Research and Data Management Center, Shenzhen, China; ^4^The Third Xiangya Hospital, Central South University, Changsha, China

**Keywords:** major public health emergencies, epidemic prevention and control policies, evolution of public opinion, sentiment analysis, LDA model

## Abstract

**Background:**

In recent years, incidents of public opinion triggered by major public health emergencies have emerged endlessly. Existing studies have focused on public attitudes during the early stages of containment measures but lacked research on how public opinion evolves after those measures are relaxed. In late 2022, however, China optimized its COVID-19 control measures, providing a unique window for this study.

**Objective:**

To reveal public attitudes toward the adjustment of response measures for major public health emergencies and how these attitudes evolve over time, and to provide a reference for improving related policies and managing public opinion.

**Methods:**

We collected Baidu Index and Weibo post data related to “epidemic prevention and control” between October 11, 2022 and March 15, 2023. Guided by the “Public Opinion Life Cycle Theory,” we analyzed the evolution of public opinion intensity using the Baidu Index. We applied the SKEP model for sentiment analysis on Weibo posts, exploring changes in public sentiment and differences among groups. Additionally, we used the LDA model for topic mining on Weibo posts, examining the evolution of discussion topics and their underlying causes.

**Results:**

During the early stages of adjustments to prevention and control measures, public opinion surged but quickly subsided to a level significantly lower than before, following the announcement of more targeted measures. In the long term, the public generally holds a positive attitude toward these adjustments, though negative sentiment may emerge in the short term. Prior to the adjustments, discussions focused on community prevention and control. In the early phase, debates were intense, with expectations for a return to normal life and economic recovery alongside concerns about health risks and medical resources. After a prolonged adjustment period, discussions on economic and daily-life topics increased, but concerns about medication and reinfection risks remained high.

**Conclusion:**

To guide the healthy development of public opinion, policymakers should clearly explain the rationale for policy adjustments, promptly address public concerns, and encourage enterprises and opinion leaders to share positive information; additionally, they should ensure sufficient medical resources are secured before implementing policy changes and roll them out in a well-organized, step-by-step manner.

## 1 Introduction

Since the 21st century, the frequency and complexity of major public health emergencies worldwide have been increasing. From SARS, H1N1 flu, Dengue fever to Ebola, MERS, Zika epidemic (ZIKV), and the recent COVID-19 epidemic, these major public health emergencies have posed a serious threat to the safety of countries around the world and have a huge impact on people’s welfare and social stability (1, 2). Preventing major public health emergencies remains a huge challenge for governments and people around the world. Since the discovery of SARS-CoV-2 at the end of 2019, many governments in countries and regions have implemented a series of public health restrictions to curb the spread of the virus, including travel restrictions, work and school suspensions, border closures, mandatory mask wearing, social distancing, and other measures to contain the spread of the virus (3). Although these policies have generally been effective in reducing the number of COVID-19 cases (4), they have had significant adverse impacts on public mental health and economic and social development (5), and policymakers have gradually recognized the need to restore normal living conditions. In order to balance epidemic prevention and economic and social development, various countries’ governments have adjusted their epidemic prevention and control policies. In May 2020, some states in the United States began to relax restrictions. As of March 2, 2022, the federal government announced its “National COVID-19 Preparedness Plan,” (6) which planned to coexist with the novel coronavirus, marking the complete release of the United States’ epidemic prevention and control policies. To effectively address the outstanding problems in the prevention and control of the novel coronavirus, the Chinese government successively issued the “Notice on Further Optimizing the Prevention and Control Measures of COVID-19” on November 11, 2022 (hereinafter referred to as the “Twenty Articles”) (7), and the “Notice on Further Optimizing the Implementation of COVID-19 Prevention and Control Measures” on December 7, 2022 (hereinafter referred to as the “Ten New Articles”) (8). This marks a significant adjustment in China’s epidemic prevention and control measures.

In the era of Web 2.0, individuals have the right to create and publish information through social media platforms, giving people more freedom and rights to express their opinions and sentiment. Since the outbreak of COVID-19, social media platforms like Twitter, Facebook, and Sina Weibo became important channels for the rapid spread of pandemic-related information, as well as for public expressions of attitudes and opinions related to epidemic prevention and control (9). The online public opinion related to the epidemic became a significant factor influencing people’s psychology, cognition, and behavior. If public opinion was not scientifically, reasonably, and effectively guided and managed, the immense panic it generates could pose a serious threat to public safety and social stability (10). Therefore, it is necessary to analyze the online public opinion related to epidemic prevention and control, helped policy makers fully understand the true attitude of the public toward epidemic prevention and control policies, and determine the mechanism of policy adjustments leading to changes in public opinion, thereby providing guidance for guiding the healthy development of public opinion. In addition, the importance of evidence related to the implementation of policies for evaluating public health policies was widely recognized (11). Evidence-based public health policy (EBPH) aimed to promote public health by making decisions based on existing data (12). Public opinion is an important reference for decision-making, so the results of public opinion analysis were also crucial for further optimizing public health policies.

At present, most studies focus on online public opinion at the initial stage of major public health emergencies and during the implementation of response policies. For example, Sukhwal et al. (13) used the BERT model to study the changes in public sentiment and focus before and after the implementation of Singapore’s COVID-19 control policy based on 240,000 posts on Facebook from January to November 2020; Chum et al. (3), based on 1.15 million tweets from March to October 2020 on Twitter, used VADER sentiment analysis method to explore the public opinion changes related to COVID-19 restrictions in Canada; Guo et al. (14) based on 100,000 posts on Sina Weibo from July 2020 to June 2021, used SnowNLP sentiment analysis and LDA theme modeling methods to analyze the evolution of public opinion in China during the COVID-19 outbreak period. The conclusions of these studies were similar. After the outbreak of the epidemic, due to the increase in the number of infected people, public sentiment rapidly decreased. However, after the government implemented restrictive measures, the epidemic was brought under control and public sentiment began to rebound.

A limited number of scholars have examined online public opinion regarding policy adjustments during the later stages of major public health emergencies. For instance, Samuel et al. (15) collected tweets related to “reopening” posted by the American public on Twitter during the first 9 days of May 2020 and conducted sentiment analysis using R’s Syuzhet package. The study found that American publics expressed more positive than negative sentiments toward COVID-19 control measure adjustments. However, this research presents several limitations. First, the study only sampled tweets from the 9 days immediately following policy adjustments, providing neither pre-adjustment baseline comparisons nor insights into long-term public attitudes. Second, the analysis focused exclusively on emotional dimensions without extracting specific viewpoints from tweet content, thus lacking depth in understanding the mechanisms driving public opinion formation. Third, the Syuzhet package’s lexicon-based approach struggles with semantically complex, lengthy texts, whereas deep learning models trained on massive text corpora could enable more precise sentiment analysis. Most importantly, the background of this study was the adjustment of epidemic control measures and the reopening of the economy in the United States at the end of April 2020. In the United States, the adjustment of epidemic control measures was a “bottom-up” process, starting from people’s protests, followed by the gradual relaxation of epidemic control measures by each state, and finally the adjustment of policies by the federal government (16). Therefore, it did not appear to be a particularly interesting finding that the American public had more positive than negative emotions toward the adjustment of epidemic prevention and control measures. China implemented the strictest and longest-lasting epidemic prevention and control measures in the world and achieved remarkable results (17, 18). The adjustment of China’s epidemic prevention and control measures was a “top-down” process, from the decision-making of the central government to the implementation by local governments. It provides a special window for studying the online public opinion related to the adjustment of response policies to major public health emergencies. However, to date, no studies have examined the evolution of online public opinion before and after the adjustment of China’s epidemic prevention and control measures at the end of 2022.

In conclusion, most of the current studies on online public opinion related to the response policies of major public health emergencies focus on the online public opinion before and after the implementation of control measures during the early stages of past epidemic outbreaks. Although some scholars have analyzed the public opinion after the adjustment of “bottom-up” epidemic prevention and control policies, there is a lack of research on the evolution of public opinion before and after the adjustment of “top-down” epidemic prevention and control policies. Moreover, there are certain limitations in the data collection, research perspective, and research methods.

Therefore, this study collected blog posts with the keyword “epidemic prevention and control” on Baidu Search Index (the world’s largest Chinese search engine) (19) and Sina Weibo (China’s largest Twitter-like social media platform) (20, 21). The collection period spanned 156 days before and after the adjustment of China’s COVID-19 epidemic prevention and control measures. By using time series analysis, sentiment analysis, and LDA topic modeling methods, this study explored the evolving characteristics of online public opinion before and after the adjustment of China’s COVID-19 prevention and control measures from three dimensions: the intensity of public opinion, the sentiment index, and the themes of public opinion. The aim was to address the deficiencies in existing research, such as the lack of attention to public opinion related to the “top-down” adjustment of epidemic prevention and control policies, the lack of comparison of public opinion changes before and after policy adjustments, the lack of observation of the public’s long-term attitude toward the adjustment of epidemic prevention and control policies, the lack of in-depth insights into the formation mechanism of public attitudes, and the difficulty of dictionary-based sentiment analysis methods in handling complex tweets.

The aims of this study were to address the following three questions:

1. What are the evolutionary characteristics and formation mechanisms of public opinion intensity before and after the adjustment of China’s epidemic prevention and control measures?
2. What are the evolutionary characteristics and formation mechanisms of public sentiment before and after the adjustment of China’s epidemic prevention and control measures?
3. What are the evolutionary characteristics and formation mechanisms of public discussion topics before and after the adjustment of China’s epidemic prevention and control measures?

Exploring the above three questions helps comprehensively reveal the impact mechanism of adjustments to response measures for major public health emergencies on the evolution of public opinion. This has important reference value for future policymakers to scientifically adjust response measures for public health events and reasonably guide the development of online public opinion.

## 2 Materials and methods

**Figure 1:**
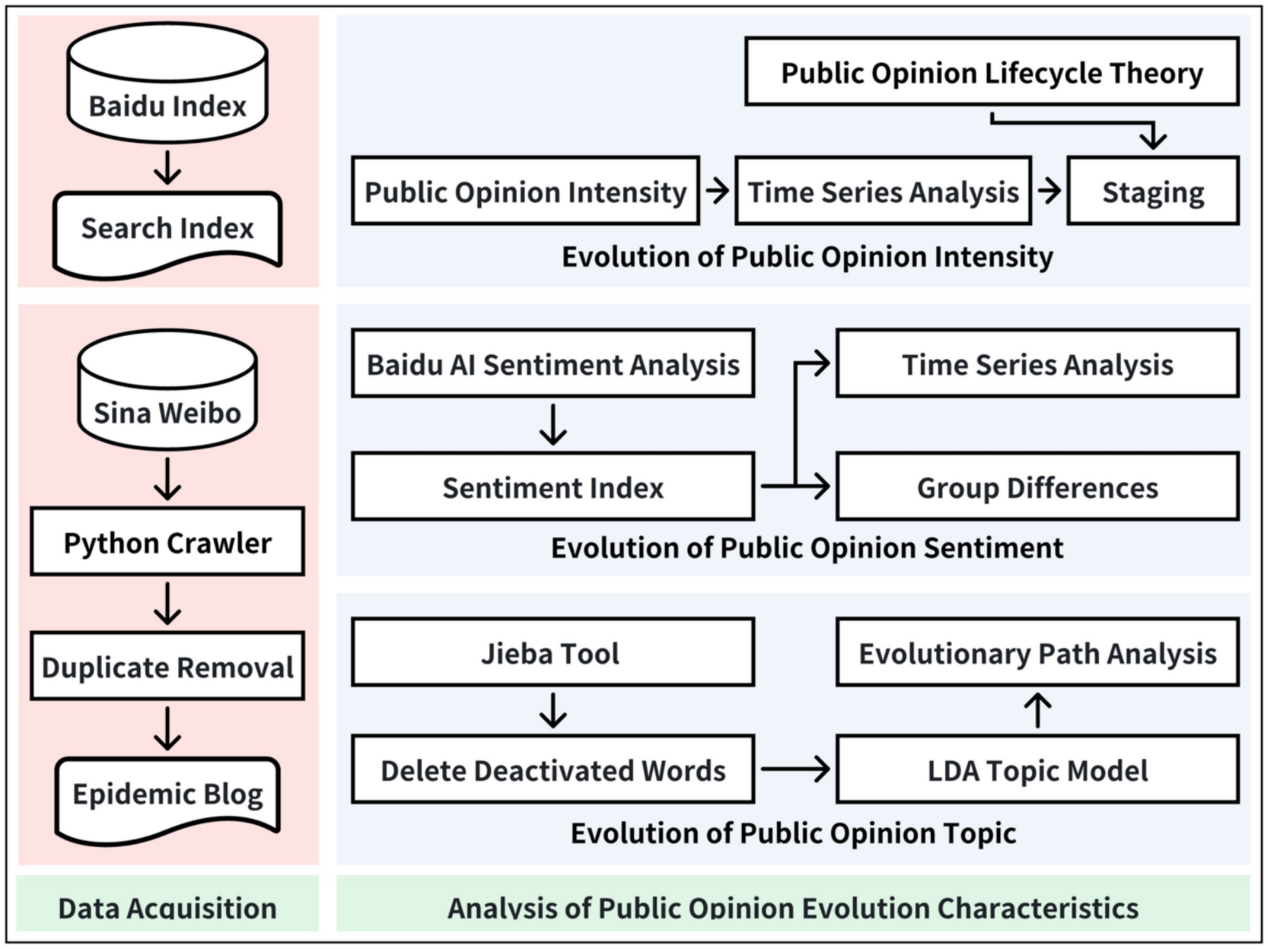
Overall research framework.

In this article, the primary research route was presented in Figure 1, consisting of five key stages. These stages are data acquisition, data preprocessing, the evolution of public opinion intensity, the evolution of public opinion sentiment, the evolution of public opinion topic.

### 2.1 Data acquisition

Baidu is the world’s largest Chinese search engine, with 632 million active users on its app in the first quarter of 2022, which is close to half of China’s total population (22). Studies have shown that there is a correlation between the vast amount of internet search data and the behavior of real-world people (23). Baidu Index is a comprehensive big data sharing service based on massive user search behavior data, which is recognized as a good indicator to measure the intensity of public opinion in China (24, 25). Therefore, this study obtained the search index data with the keyword “epidemic prevention and control” from the Baidu Index platform during the period from October 11, 2022 to March 15, 2023, and analyzed the evolution of the intensity of online public opinion before and after the adjustment of China’s epidemic prevention and control measures.

Sina Weibo is China’s largest and most popular social media platform, with 584 million active users on the Weibo platform as of September 2022 (14). Users can publish posts on Weibo without censorship, and these posts are allowed to freely express views on policies and special issues (26, 27). The opinions of Weibo users largely represent Chinese public opinion, and most studies on Chinese online public opinion are also based on Weibo data (28, 29). Therefore, it is reasonable for this study to choose Weibo as the data source.

Using a self-developed Python web crawler program, we scraped the first 50 pages of Weibo posts related to “epidemic prevention and control” published by individuals and groups on the Sina Weibo platform every day from October 11, 2022, to March 15, 2023. This is because Weibo only displays the first 50 pages of posts, and we cannot obtain all of them. Since the focus of this study is public opinion, and content posted by Chinese government media typically reflects policy rather than individual perspectives, posts from government media were not collected (30). The dataset includes nine fields: “username,” “post content,” “post time,” “IP location,” “user type,” “gender,” “reposts,” “comments” and “likes” for a total of 57,382 data entries. Since users who publish an extremely high number of tweets only reflect a minority opinion (31), we used “username” and “post content” fields to remove duplicates, leaving 51,972 valid data entries.

To study the impact of the pandemic on public opinion, we obtained daily data on new confirmed COVID-19 cases and asymptomatic infections from the website of the National Health Commission of China for the period from October 11 to December 24, 2022. Since the Chinese government stopped releasing daily infection and case data on December 25, 2022, we were unable to obtain information beyond this date (32).

### 2.2 Data preprocessing

In the first step, the Jieba tool (a commonly used Chinese word segmentation system based on machine learning) was used to segment the Weibo posts in Chinese.

In the second step, stop words were removed. Stop words are frequently occurring words in a text that lack substantial meaning or are semantically ambiguous, such as “de,” “a,” “ya,” as well as various punctuation marks and interjections in Chinese. The high frequency of these words can interfere with the extraction and analysis of key information from the text. To more accurately identify the main themes of the text, this study used the “Harbin Institute of Technology Stop Words List” to filter out meaningless words from Weibo comments. This list is a commonly used resource in the field of Chinese natural language processing and contains 746 Chinese stop words (33).

### 2.3 Analysis of the evolution of public opinion intensity

Regarding the division of development stages of online public opinion, different scholars have different bases and methods. American scholar Horton, borrowing the concept of the biological life cycle, first proposed the concept of the information life cycle, similar to the life cycle path, in 1985. He divided the information resource life cycle into four stages: birth, growth, decline, and death (34). Subsequently, Fink, S. divided public opinion communication into four stages based on this, namely the incubation period, outbreak period, spread period, and recovery period, preliminarily forming the “public opinion life cycle theory” (35).

Therefore, this study utilized Baidu Search Index as a metric to gauge the intensity of public opinion related to epidemic prevention and control in China. Guided by the “public opinion lifecycle theory,” and in conjunction with the timing of adjustments to China’s epidemic prevention and control measures, the study divided the development process of public opinion into several stages and explored its evolutionary characteristics through time series analysis.

### 2.4 Analysis of the evolution of public opinion sentiment

Sentiment analysis is an important branch of natural language processing research, which can extract sentiment information from a large amount of unstructured text and has been widely used to mine human sentiment tendencies from posts on Twitter, Facebook, and other social media (36, 37).

In current research on the sentiment analysis of review texts, the sentiment analysis methods used generally fall into two categories. One category is the dictionary-based sentiment analysis method, such as the Syuzhet package in the R language and the SnowNLP library in Python. Methods of this kind are based on the word-sentiment mapping of a fixed dictionary and are only suitable for the sentiment analysis of simple short texts. It is difficult for them to handle long texts with complex semantics (14, 15). The other category of sentiment analysis methods is deep learning models trained on massive corpora, such as the BERT pre-trained model. Methods of this kind can identify the complex semantics in long texts and have significant advantages over dictionary-based sentiment analysis methods (13).

SKEP (Sentiment Knowledge Enhanced Pre-training for Sentiment Analysis) is a sentiment pre-training model based on sentiment knowledge enhancement proposed by Baidu’s NLP research team in 2020. It uses an unsupervised method to automatically mine sentiment knowledge, and then utilizes the sentiment knowledge to construct pre-training objectives, enabling the model to learn to understand sentiment semantics. SKEP provides a unified and powerful sentiment semantic representation for various sentiment analysis tasks. Existing research has shown that in the task of Chinese text sentiment analysis, the SKEP model outperforms the BERT model developed by Google (38).

Therefore, in this study, the SKEP model developed by Baidu was used to analyze the sentiment tendency in Weibo texts. By calling the API, the probabilities of positive tendency, negative tendency, and the confidence level were returned. To ensure the reliability of the sentiment analysis results, only 21,682 data with an sentiment analysis confidence of 90% or higher were selected as research samples. Then, we analyzed the evolution characteristics of sentiment tendencies, and analyzed the differences and reasons for different user types (ordinary users, senior users, celebrities, and enterprises) and different gender groups’ attention to adjustments in epidemic prevention and control measures.

### 2.5 Analysis of the evolution of public opinion topic

Topic modeling is an unsupervised machine learning technique that uses statistical probabilities and the correlation between words to uncover abstract topics hidden within one or multiple documents (33). LDA is currently the most widely used topic model, which estimates the relationship between topics and words by assuming “a topic is composed of a mixture of words, and a document is composed of a mixture of topics” (39).

Therefore, this study used the LDA model to uncover the topics discussed in Weibo posts at each stage of public opinion. Two key metrics for evaluating a topic model are perplexity and coherence (40). The perplexity metric measures the uncertainty of the topic model’s prediction of the topic probability distribution for documents. A lower perplexity value indicates less uncertainty in predicting the topic of new documents, signifying a better fit of the model. The coherence metric assesses whether the topics are internally consistent by calculating the similarity between words within a topic. Higher coherence suggests that the generated topics are clearer and more understandable. Therefore, when determining the number of topics for a model, it is necessary to consider both perplexity and coherence measures simultaneously. The goal is to select a number of topics that exhibit lower perplexity and higher coherence to balance the goodness of fit and interpretability of the topic model. Additionally, having too many topics may lead to overfitting, so it is important to avoid setting an excessive number of topics. After identifying the themes of each stage in public opinion analysis, calculated the cosine similarity between the themes of adjacent stages and create a Sankey diagram to visualize the topic evolution. This will further enable the analysis of the characteristics of public opinion topic evolution.

This study used the Gensim library in Python to build the LDA model and calculate perplexity and coherence scores, and employed the ‘cosine_similarity’ method from the sklearn library to calculate the cosine similarity between topics.

## 3 Results

### 3.1 The evolution of public opinion intensity

**Figure 2:**
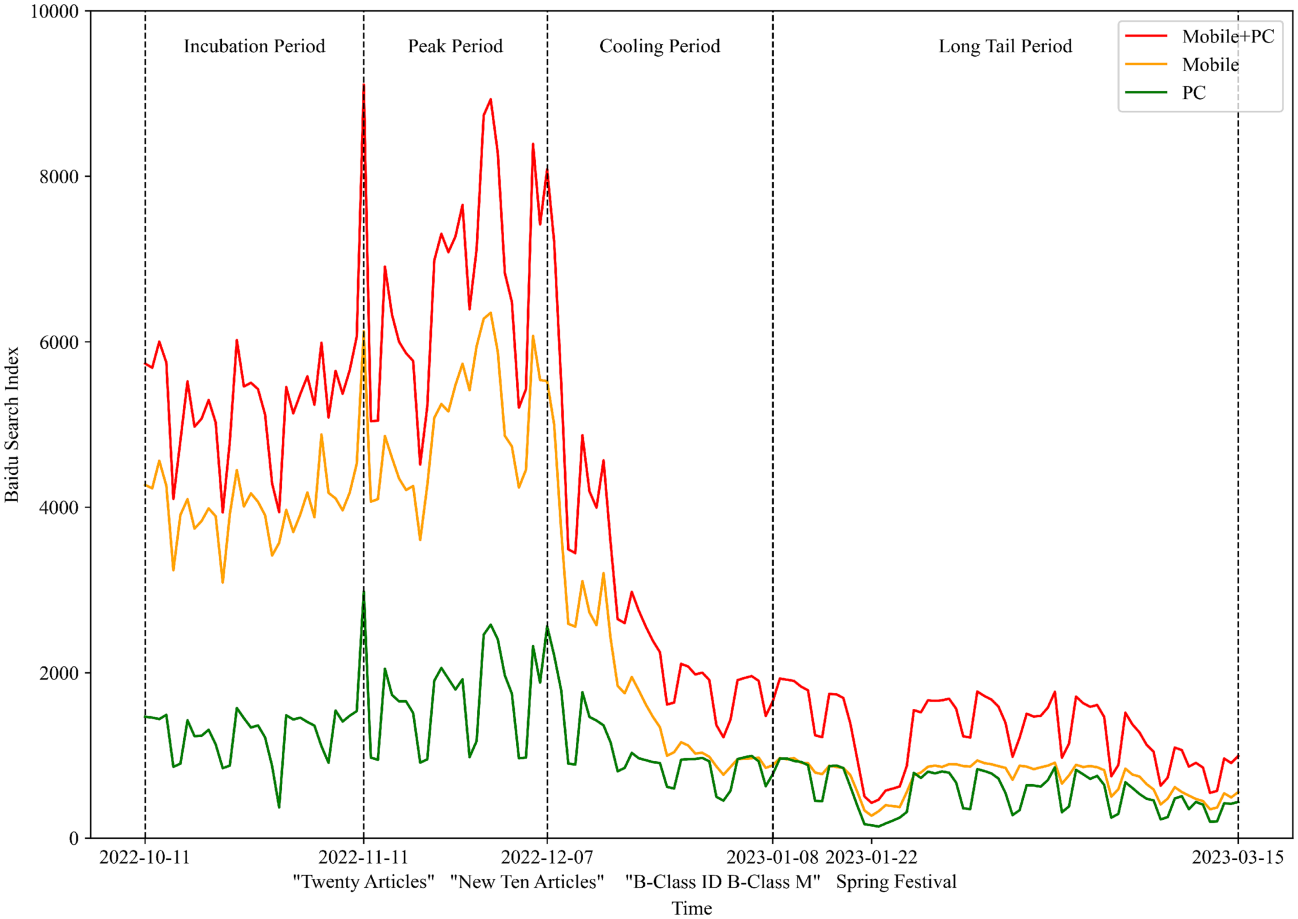
Evolution of public opinion intensity over time.

The evolution process of the intensity of public opinion before and after adjusting the COVID-19 prevention and control measures in China is illustrated in Figure 2. From October 11, 2022, to November 10, 2022, in the month preceding the adjustment of China’s pandemic prevention measures, the intensity of public opinion related to “pandemic prevention” remained at a high level due to the gradual increase in new infections and the implementation of control measures. On November 11, 2022, the “Twenty Articles” were released, marking the first significant document from the Chinese government that aimed to optimize pandemic prevention measures. This document instructed local governments not to impose lockdowns arbitrarily, not to prolong lockdowns indefinitely, and generally not to conduct mass nucleic acid testing. The public had intense discussions about the adjustments to the prevention and control measures. At the same time, due to these adjustments, the number of infections rapidly increased during this period, leading to a swift rise in the intensity of public opinion. On December 7, 2022, the “New Ten Articles” were issued, mandating precise determination of risk areas, further reduction of the number of people under lockdown, and ensuring the normal operation of society. Following this, the intensity of public opinion began to decline rapidly. On January 8, 2023, the Chinese government announced the reclassification of COVID-19 from a “Category B infectious disease managed as Category A” to a “Category B infectious disease managed as Category B” with the core content being the cessation of risk area delineation and quarantine measures. By this time, public opinion had decreased to a low level, and entered a phase of gradual decline thereafter. The evolution of public opinion aligns with the four stages of the “public opinion life cycle theory”—incubation period, outbreak period, spread period, and recovery period. Given that this public opinion was directly influenced by policy adjustments, this study uses the timing of the three policy adjustments as the nodes for dividing the stages of public opinion development. Thus, the development of public opinion can be divided into four stages: the “Incubation Period” (October 11, 2022, to November 10, 2022), the “Peak Period” (November 11, 2022, to December 6, 2022), the “Cooling Period” (December 7, 2022, to January 7, 2023), and the “Long Tail Period” (January 8, 2023, to March 15, 2023).

**Figure 3:**
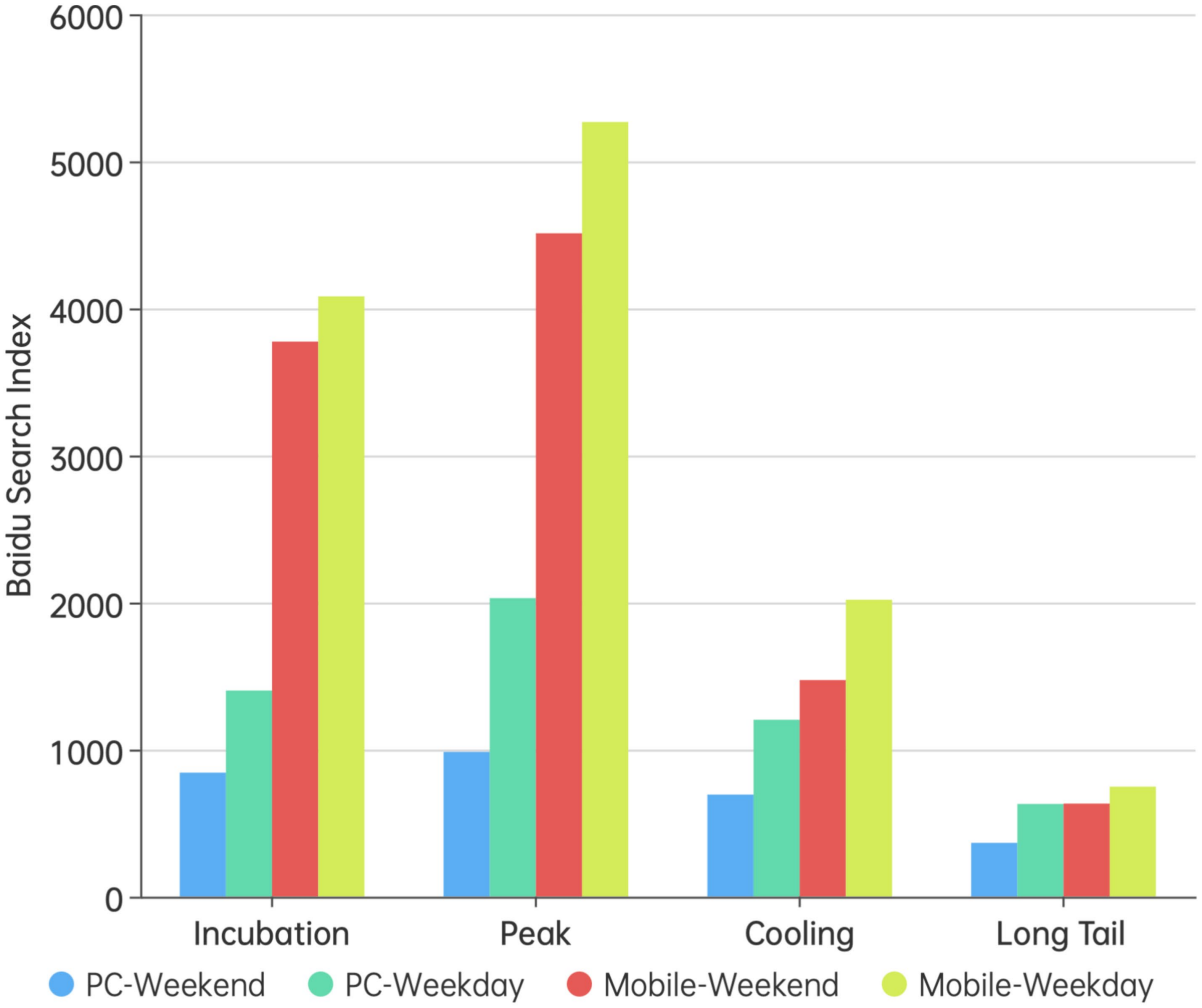
Difference in public opinion intensity between weekends and weekdays at various stages of public opinion.

It was worth noting that around January 22, 2023, during the Chinese traditional Spring Festival, public attention to epidemic prevention and control decreased significantly. Further observation revealed that the declines in public sentiment tended to occur in pairs and these points corresponded exactly with weekends. To investigate whether there was a difference in the intensity of pandemic prevention and control sentiment between weekdays and weekends, a grouped bar chart of sentiment intensity during different stages on weekdays and weekends was created (as shown in Figure 3). The analysis results show that, whether on PC or mobile, the search index for “pandemic prevention and control” was lower on weekends than on weekdays. An independent t-test using SPSS 27 revealed that this difference was very significant on PC (*p* < 0.01), but was not significant on mobile. This phenomenon can be attributed to two factors: first, during weekdays, people have an increased need to travel due to work or school, thereby paying more attention to pandemic prevention and control; second, compared to weekends, people typically need to use computers for work on weekdays, thus increasing the likelihood of obtaining information on PC. In summary, the evolution of online public opinion related to pandemic prevention and control is mainly influenced by policy factors and is also closely related to holidays and other major social events.

Another noteworthy phenomenon was that during the latent and peak phases of public opinion, the search index for mobile devices was significantly higher than for PC. However, during the cooling phase, the gap between these two began to narrow gradually. By the long-tail phase, the difference in the search index between mobile devices and PC had become very minimal. Generally speaking, mobile devices were more convenient for obtaining information compared to PC. This phenomenon indicated that during the lockdown phase and the early stages of lockdown adjustments, the public’s demand for information related to pandemic lockdowns was particularly urgent. However, after the lockdown adjustments had been in place for some time, although the public still maintained an interest in lockdown information, the level and urgency of their attention had significantly decreased.

### 3.2 The evolution of public opinion sentiment

**Figure 4:**
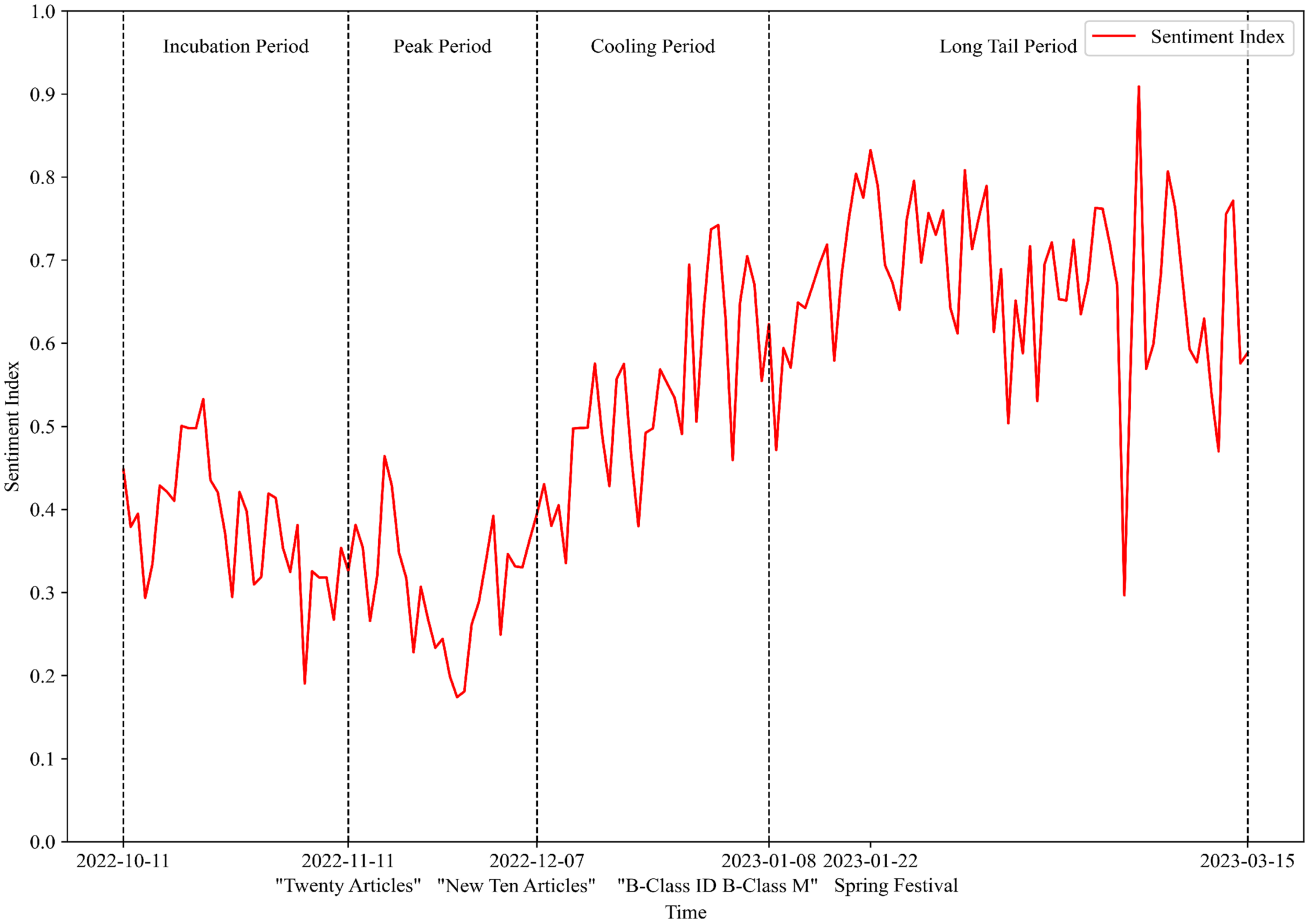
The evolution of public opinion sentiment over time.

Using a pre-trained SKEP model from Baidu, the sentiment index of 51,972 Weibo posts was analyzed. From these, 21,682 posts with a prediction confidence above 0.9 were selected to plot the sentiment index trend related to “pandemic prevention and control” (as shown in Figure 4).

As indicated by the figure, before the release of the “Twenty Articles” due to the stringent lockdown phases, the public sentiment index was generally below 0.5 and showed a fluctuating downward trend. Following the release of the “Twenty Articles” there was a brief initial increase in the sentiment index, but it subsequently declined to lower levels. This change suggested that public attitudes were momentarily optimistic upon the announcement of the pandemic control adjustments. However, as the intensity of the lockdowns decreased, the infection rates rapidly increased, and the symptoms of post-infection began to emerge, leading to a shift toward negative public sentiment.

After a period of adaptation, as more infected individuals recovered, the public sentiment index began to show a fluctuating upward trend. The release of the “New Ten Articles” further optimized control measures, and the social and economic order gradually returned to normal. Correspondingly, the public sentiment index also fluctuated upward to higher levels. Following the announcement of the “Category B infectious disease managed as Category B” policy, social life had essentially returned to the pre-pandemic state, and the economy had begun to grow steadily. During this phase, the public sentiment index fluctuated between 0.6 and 0.8, which indicated a generally positive public response to the long-term adjustments in pandemic control measures.

**Figure 5:**
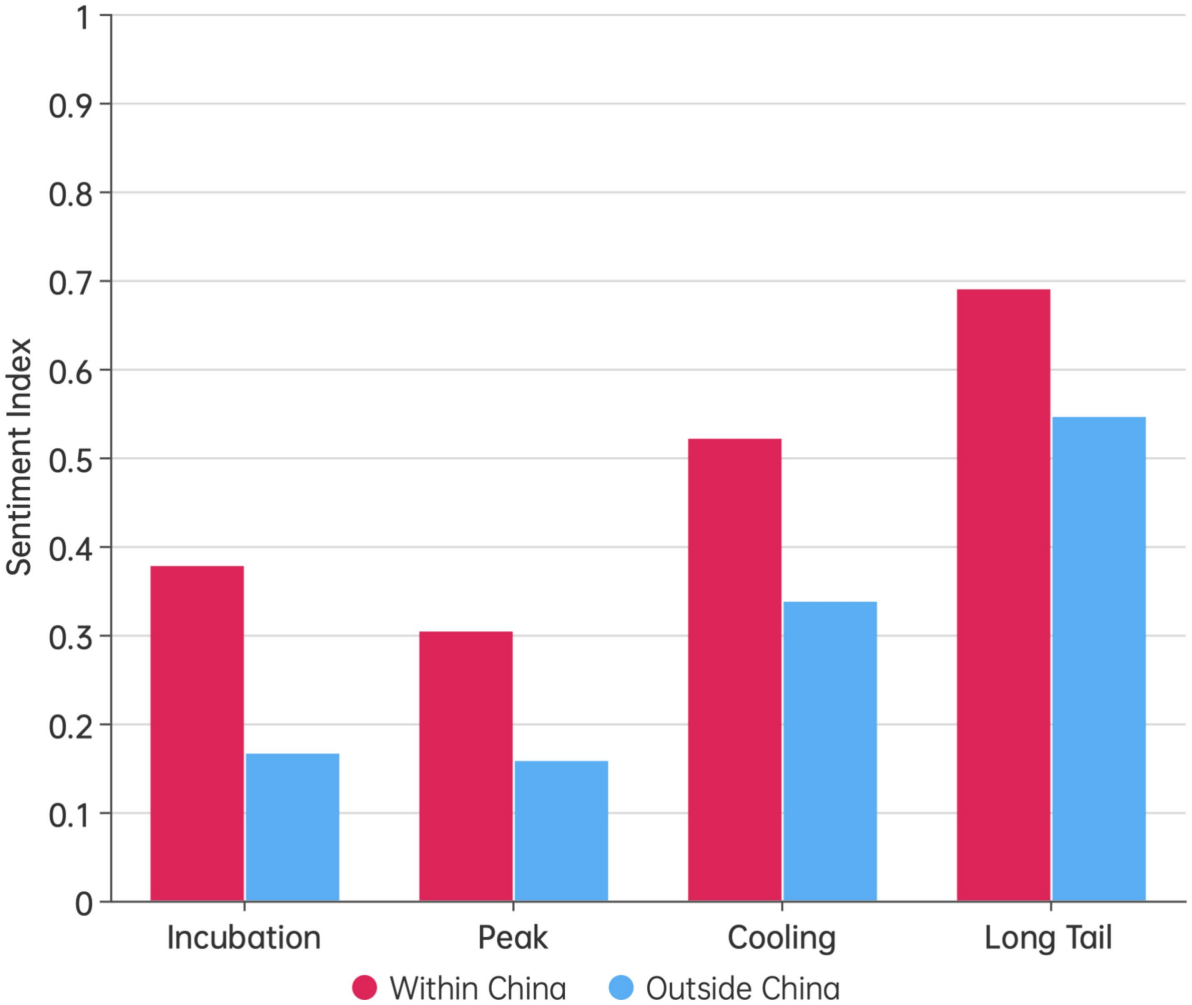
The differences in public opinion sentiment between domestic and overseas audiences in China at various stages of public opinion.

Figure 5 compared the average sentiment index of Weibo posts with IP addresses located within China and outside of China across different public opinion stages. The figure showed that at each stage of public opinion, the sentiment index of domestic posts was significantly higher than that of overseas posts, as confirmed by an independent t-test (*p* < 0.05). Notably, the sentiment index during the cooling period for posts outside of China was similar to that during the peak period for posts within China. Additionally, the sentiment index during the long-tail period for posts outside of China was relatively close to that during the cooling period for posts within China. This phenomenon indicated a certain lag in the public’s perception outside of China regarding the country’s pandemic prevention and control measures.

**Figure 6:**
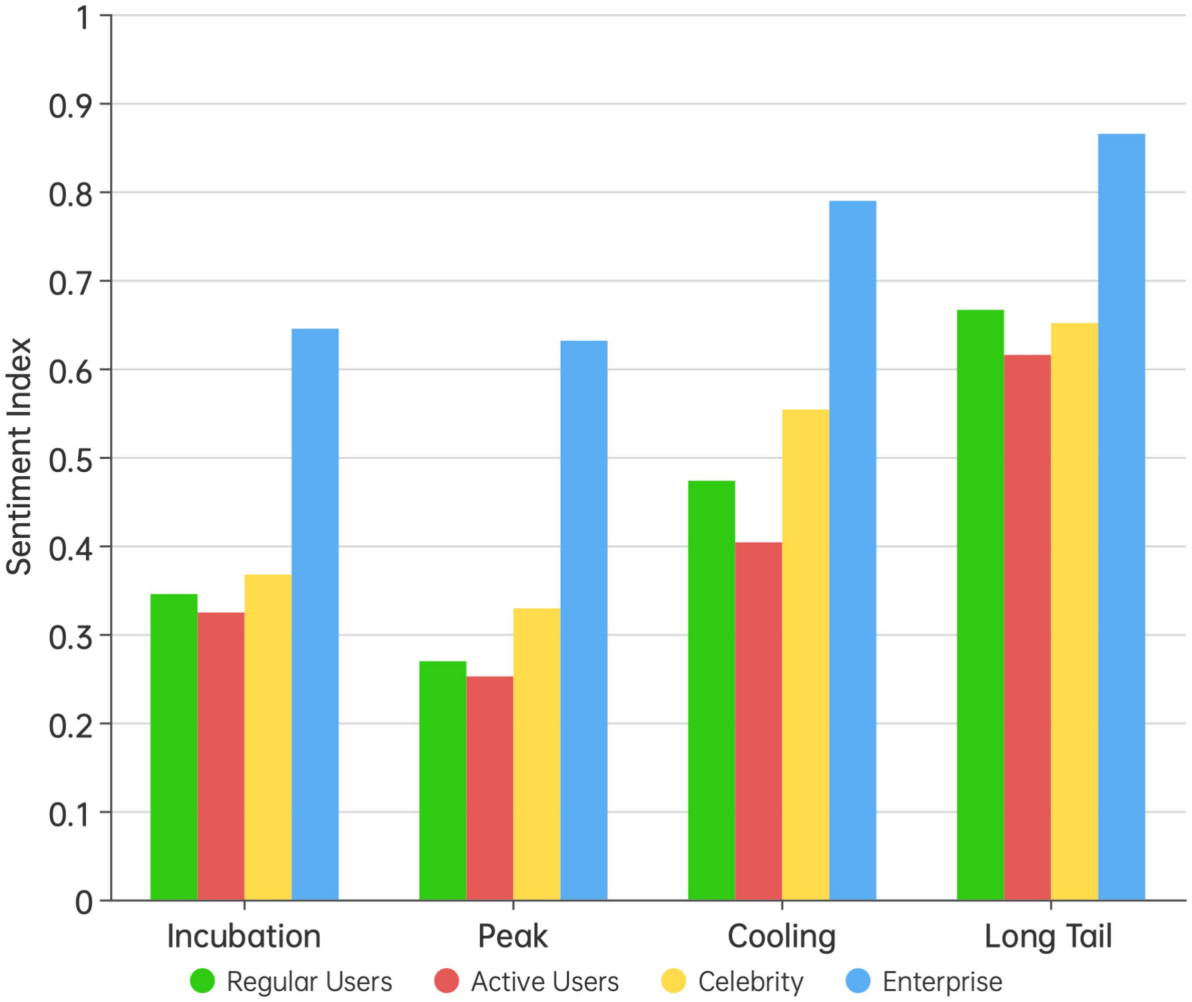
The differences in public opinion sentiment among different groups at various stages of public opinion.

Figure 6 compared the sentiment index of different types of users at various stages of public opinion. Active users consistently had lower sentiment indices than regular users at all stages. Celebrities, except during the long-tail phase where their sentiment index was slightly lower than that of regular users, have higher sentiment indices than regular users at all other stages. However, independent t-tests indicated that the differences between these groups were not statistically significant (*p* > 0.05). In contrast, enterprise users’ posts exhibited significantly higher sentiment indices than those of individual users at all stages, and independent t-tests confirmed that this difference was significant (*p* < 0.05).

**Figure 7:**
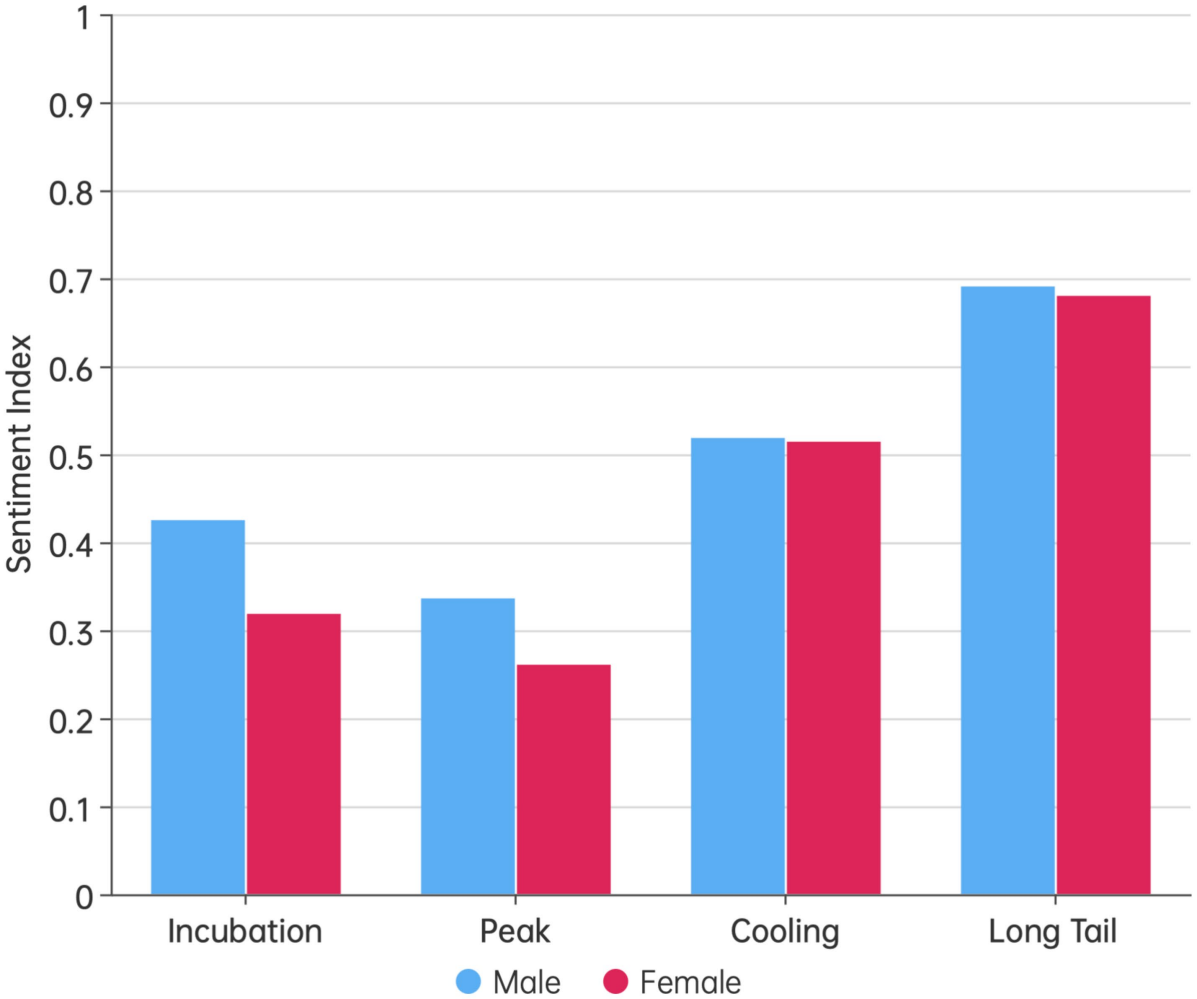
The differences in public opinion sentiment between men and women at various stages of public opinion.

Figure 7 compared the sentiment index differences between male and female users across various stages of public opinion. At each stage, men exhibited a more positive attitude toward pandemic prevention and control than women. However, the extent of this difference varied across the stages. During the latent and peak phase, there was a significant difference in the sentiment indices between men and women (*p* < 0.05). In the cooling and long-tail phase, although men’s sentiment indices were still higher than women’s, the differences were not statistically significant (*p* > 0.05).

### 3.3 The evolution of public opinion topic

**Figure 8:**
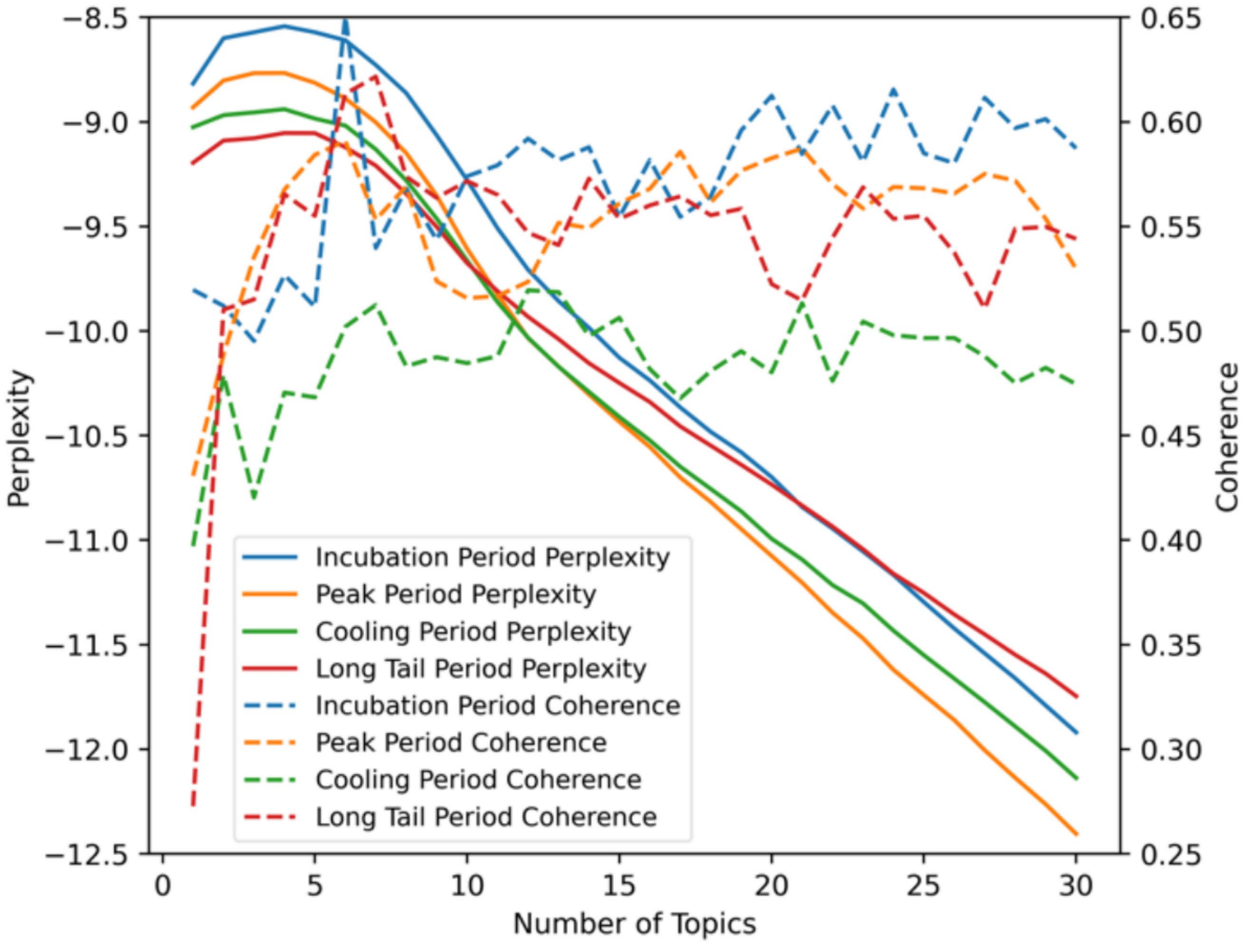
Perplexity and coherence curves.

This study employed the LDA model to explore topics related to “epidemic prevention and control” in Weibo posts across different stages. The effectiveness of topic extraction was evaluated using two metrics: perplexity and coherence, to determine the optimal number of topics. To prevent overfitting, it was essential to avoid setting too many topics. Therefore, the perplexity and coherence curves for LDA models with topic numbers ranging from 1 to 30 were plotted, as shown in Figure 8. Using the incubation period as an example, the solid blue line represented the perplexity curve. When the number of topics was less than 5, the perplexity showed an increasing trend. Subsequently, as the number of topics increased, the perplexity continuously decreased. The dashed blue line represented the coherence curve, which first showed a wave-like increase, followed by a wave-like decrease. When the number of topics was 6, coherence reached a peak, but the perplexity was still relatively high, making it unsuitable as the optimal number of topics. However, when the number of topics reached 24, coherence peaked again, and the perplexity had dropped to a relatively low level. Therefore, the optimal number of topics for the incubation period LDA model was set to 24. Similarly, for the peak, cooling, and long-tail period LDA models, the optimal number of topics was set to 21, 21, and 23, respectively, based on a comprehensive consideration of perplexity and coherence. This ensured that the models maintained high accuracy while retaining good interpretability.

**Figure 9:**
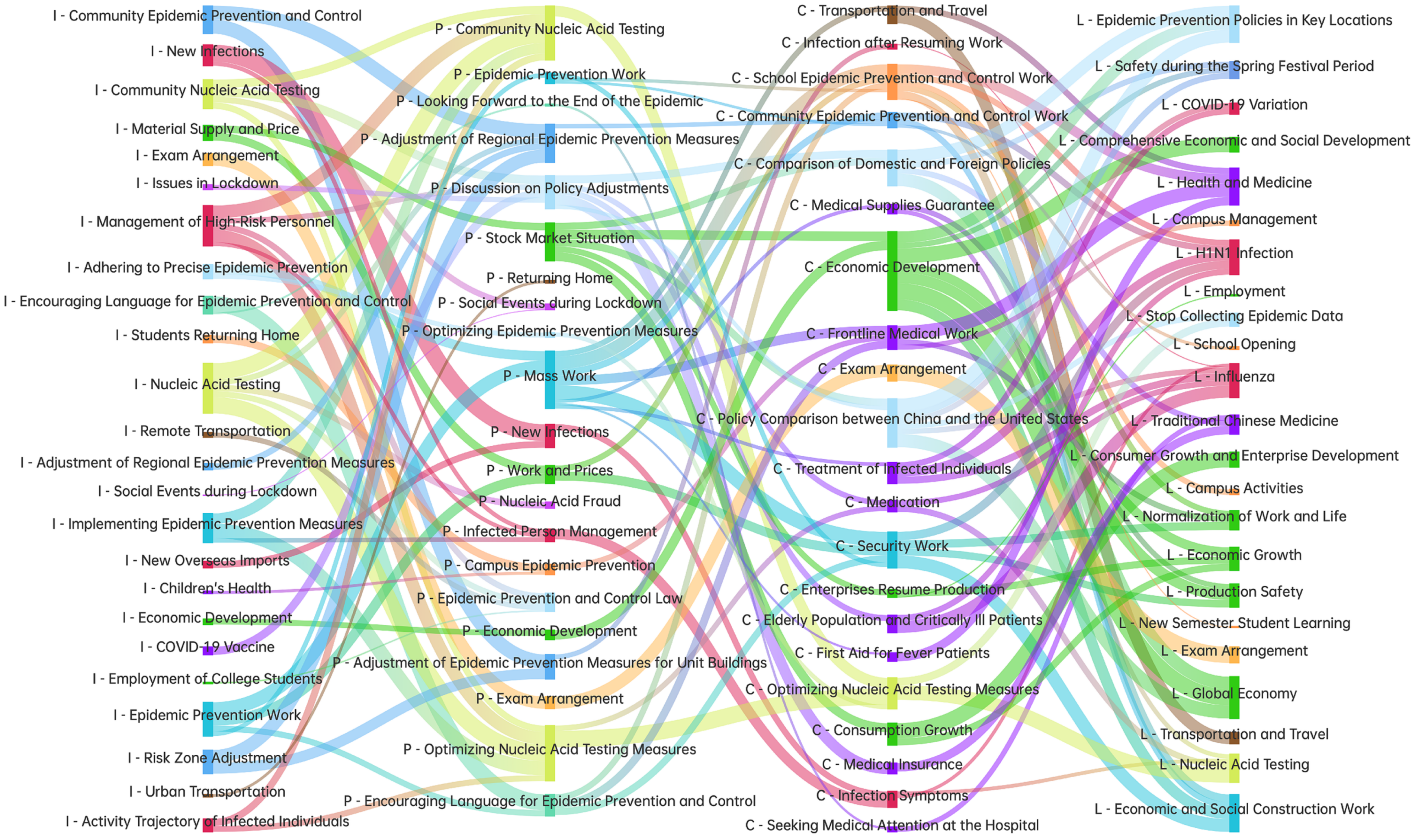
Topic evolution path.

To explore the evolutionary path of topics, the cosine similarity between topics at adjacent stages was calculated, and a Sankey diagram of topic evolution was drawn, as shown in Figure 9. The four columns in the figure, from left to right, corresponded to the “Incubation Period” “Peak Period” “Cooling Period” and “Long-tail Period” respectively. The prefix of the topic names corresponded to the abbreviation of the stage they belonged to. Within each column, the topics were arranged from top to bottom in order of their intensity, with the highest intensity appearing at the top. The colors of the topics were used to distinguish different types. Red represented topics related to the virus and infection, yellow to nucleic acid testing, orange to students and examinations, green to the economy, blue to epidemic prevention policies and work, and purple to medical care.

The number of various types of topics at each stage of public opinion development was statistically analyzed, as shown in Table 1. Consistent with Figure 9, different colors were used to represent different topic categories, while variations in color intensity were employed to indicate the number of topics. This visual encoding enables a clear and intuitive illustration of the changes in topic distribution across different stages of public opinion evolution.

**Table 1:** The number of various types of topics at various stages of public opinion.

Topic types	Incubation period	Peak period	Cooling period	Long-tail period
Viruses and infections	4	2	2	3
Economy	3	3	3	8
Regional epidemic prevention and control	3	2	1	0
Epidemic prevention and control work	3	4	1	1
Problems in lockdown	2	2	0	0
Nucleic acid testing	2	2	1	1
Transportation	2	1	1	2
Education	2	2	2	5
Medical care	2	0	8	2
Encouraging words for fighting the epidemic	1	2	0	0
Policy-related discussions	0	1	2	2

Figure 9 illustrates that “Community Epidemic Prevention and Control” was the most prominent topic during the incubation period of public opinion. This topic, in combination with “Adjustment of Regional Epidemic Prevention Measures” and “Risk Area Adjustment,” evolved into the peak period topics of “Adjustment of Regional Epidemic Prevention Measures” and “Adjustment of Epidemic Prevention Measures for Unit Buildings.” These topics are all related to regional epidemic prevention and control and were therefore categorized under the “Regional Epidemic Prevention and Control” topic type in Table 1. During the cooling period, the number of such topics decreased to one, and during the long-tail period, they disappeared entirely. This indicates that public attention was primarily focused on community-level epidemic prevention during the implementation phase of control measures. As lockdown measures were gradually lifted, public concern regarding community epidemic prevention declined accordingly. Following the complete removal of lockdown restrictions, regional epidemic prevention and control ceased to be a focus of public interest.

During the incubation period, “Viruses and Infections” was identified as one of the primary topics of public concern, with four topics classified under this category: “New Infections,” “Management of High-Risk Personnel,” “New Overseas Imports” and “Activity Trajectory of Infected Individuals.” During the peak period, the number of topics in this category was reduced to two—namely, “New Infections” and “Infected Person Management”—indicating a decline in public attention to viral infections. Nevertheless, data revealed that, as preventive measures were adjusted, infection counts actually increased sharply. This pattern suggested that, when case numbers were relatively low, public concern was concentrated on who had been infected and on how infected individuals could be effectively managed. In contrast, once case counts rose to levels at which any individual could potentially become infected, the identification and management of infected persons ceased to be the primary focus of public concern. During the cooling period, the number of topics remained at two but evolved from the peak-period topics of “New Infections” and “Infected Person Management” to “Infection after Resuming Work” and “Symptoms of Infection.” This shift reflected that, following the adjustment of lockdown measures, concerns had emerged regarding SARS-CoV-2 infection during the resumption of work and increased social interactions, as well as regarding the symptoms manifesting post-infection. In the long-tail period, these topics further evolved into “COVID-19 Variation,” “H1N1 Infection” and “Influenza.” This evolution indicated that, after experiencing the discomfort associated with COVID-19 infection, public attention had shifted toward worries about reinfection due to novel viral variants and toward developments in other epidemics such as H1N1 and influenza.

In the incubation period, the topic of “nucleic acid testing” was one of the issues receiving notable public attention, and its intensity rose to its maximum during the peak period. This suggests that, as infection numbers increased, community-based nucleic acid testing was conducted more frequently. Moreover, following the release of the “Twenty Articles,” which required local governments to optimize nucleic acid testing procedures, the topic “Optimizing Nucleic Acid Testing Measures” emerged. During the cooling period, the number of nucleic acid testing–related topics decreased from two to one, and topic intensity was substantially reduced. This reduction can be attributed to the further optimization of epidemic prevention measures enacted under the “New Ten Articles,” which greatly diminished the need for nucleic acid testing. By the long-tail period, topic intensity had declined even further.

Economic issues were a topic of public concern across all stages of public opinion, but the focus of that concern differed by stage. During the incubation period, the topic “Material Supply and Price” received the highest level of public attention, whereas “Economic Development” attracted relatively limited attention. In the peak period, however, attention to price-related topics decreased significantly, while the topic “Stock Market Situation” was highly prominent. During the cooling period, a sharp increase was observed in public attention to “Economic Development.” By the long-tail period, “Economic Development” had evolved into “Comprehensive Economic and Social Development,” and additional topics—namely, “Consumer Growth and Enterprise Development,” “Global Economy,” and “Employment”—emerged, increasing the number of economic topics from three to eight. It was indicated by these evolutionary patterns that during the lockdown, the public had been more concerned with the supply and price of goods. As lockdown measures were adjusted, a gradual shift in public attention toward macroeconomic development was observed.

Education-related topics were present across all stages of public opinion evolution. During the incubation period, the “Exam Schedule” and “Students Returning Home” topics were observed to have high intensity. This phenomenon was attributed to the fact that, during lockdowns, schools were closed and students were sent home to prevent cluster infections, leading to heightened public concern regarding students’ return home and examination arrangements. In the peak period, however, the intensity of education-related topics was found to have declined sharply, as the majority of students were resting at home. Upon entering the cooling period, following the release of the “New Ten Articles,” which mandated the resumption of in-person teaching, the intensity of education-related topics increased rapidly, the “School Epidemic Prevention and Control Work” topic exhibited the highest intensity, followed by the “Exam Arrangement” topic. This indicated that, after adjustments to epidemic prevention measures, the public’s primary concern pertained to on-campus epidemic prevention measures, followed by examination arrangements. In the long-tail period, the number of education-related topics expanded from two to five, evolving into new topics such as “School Starts,” “Campus Activities” and “New Semester Student Learning,” thereby demonstrating that, over the extended time frame after the adjustment of epidemic prevention measures, the public continued to focus on children’s health and learning within schools.

The “Policy-related Discussions” topic category captured public discourse on epidemic prevention policies. During the incubation period, given that China had already implemented epidemic control measures for nearly 3 years, the public was very familiar with these policies and measures. Consequently, no topics in this category were identified, indicating that public discussion of epidemic prevention policies was not prominent. However, in the peak period—following the release of the “Twenty Articles”—a topic entitled “Discussion on Policy Liberalization” emerged with high intensity. As shown in Figure 9, this topic was closely associated not only with the incubation-period topics “Community Nucleic Acid Testing,” “Issues in Lockdown” and “COVID-19 Vaccine,” but also with the “Medical Care” topic that appeared in the cooling period. This pattern suggests that the public held dual considerations regarding adjustments to epidemic prevention measures. On the one hand, previous control measures had, to some extent, disrupted production and daily life, and the public hoped that policy adjustments would alleviate these burdens. On the other hand, the public was deeply concerned about the health impacts of such adjustments—particularly the increased demand for medical supplies resulting from rising infection numbers, as well as the need to ensure the safety of older adult and critically ill patients. It was also argued that the prior widespread administration of COVID-19 vaccines could confer a degree of immune protection, thereby mitigating excessive concern. Upon entering the cooling period, the “Discussion on Policy Liberalization” topic evolved into two distinct subtopics—“Domestic and Foreign Policy Discussions” and “Comparison of Policies between China and the United States”—reflecting the public’s engagement in comparative and in-depth exploration of epidemic prevention measures both at home and abroad.

The “Epidemic Prevention and Control Work” topic represents public commentary on government efforts to prevent and control the pandemic. This topic evolved dynamically across successive epidemic stages: during the incubation period, the “Adhere to Precise Epidemic Prevention” topic was most salient; it then shifted to the “Mass Work” topic in the peak period; thereafter, in the cooling period, the “Security Work” topic predominated; and, finally, in the long-tail period, the focus moved to the “Economic and Social Construction Work” topic. These transitions indicate that, before policy adjustments, the public’s attention was directed toward the government’s precision in epidemic prevention. In the initial stage of policy adjustment, attention was redirected to the government’s execution of newly implemented policies. Subsequently, as lockdown measures were modified and infection counts rose, public concern was focused on the government’s efforts to secure medical supplies. In the later phase of policy adjustment, the public’s attention was ultimately directed toward the government’s role in guiding economic and social recovery.

During the cooling period, eight healthcare-related topics—including “Medical material security,” “Frontline medical work” and “Treatment of infected individuals”—were suddenly observed. This emergence indicates that, as infection counts increased, public demand for medical services rose sharply, rendering the allocation of medical resources a focal point of concern. Upon entering the long-tail period, these topics were consolidated into two overarching topics: “Health and Medicine” and “Traditional Chinese Medicine.” This transition reflects that, following recovery from infection, the demand for immediate medical care declined; however, compared with the period prior to adjustments in epidemic prevention measures, public attention to health was markedly elevated.

## 4 Discussion

### 4.1 Principal findings

The fluctuation in public opinion intensity was mainly influenced by pandemic control measures. During lockdown phases, the intensity of public opinion is high. When new control measures were announced, the intensity rose even higher initially. However, it could quickly decline once more specific and clear policies were released. Public opinion trends were also related to holidays, with a significant drop in intensity on weekends and major holidays concerning pandemic control. During lockdowns and the initial stages of policy adjustments, the public’s demand for information related to the pandemic was more urgent, leading to increased use of mobile devices to access or share information.

Public sentiment was also primarily influenced by pandemic control measures. During lockdowns, the disruption to daily life and work led to a gradual decline in sentiment. After the announcement of policy adjustments to these measures, there was a brief surge of excitement among the public. However, as infection rates rose and symptoms appeared, sentiment began to turn negative. Over time, as symptoms subsided and normalcy returned, public sentiment gradually rose to levels significantly higher than those before the adjustments. In the long term, the adjustments were well-received by the public, but the negative sentiment that surfaced during the transition needed active guidance. Outside of China, delayed and sometimes partial access to information about China’s pandemic control efforts led to relatively lower sentiment. Sentiment among corporate users was notably more positive than that of individuals, and celebrities’ sentiment was slightly more positive compared to that of ordinary people. Due to various cultural, social, and economic adversities, women were more likely to report mental health problems than men in epidemic situations, and were more willing to disclose their suffering (41). During lockdowns, women’s sentiment was more negative than men’s, but this difference gradually diminished with the adjustment of control measures.

Overall, the public held a dual perspective on the adjustment of control measures: on one hand, there was an expectation to adjust these measures to restore normal work and life order; on the other hand, there is was concern that the adjusted measures may increase the risk of infection, posing a threat to the health of themselves and their families. From a long-term perspective, the mainstream view among the Chinese public was a desire to return to normal life. Specifically, during the lockdown phase, the public was most concerned about community epidemic prevention measures, especially the arrangements for nucleic acid testing. The number of new infections and the management of infected individuals were also focal points of public attention. Additionally, the public voiced opinions on issues such as the supply and price fluctuations of goods during epidemic prevention. In the early stages of adjusting control measures, public attention to the number of infections and management measures significantly decreased, as did concerns over prices and other prevention-related issues. The focus shifted to the adjustment of control measures, official explanations of these adjustments, and changes in regional control measures. At the same time, public attention to economic development began to increase. As control measures were further adjusted and work and study gradually resumed, the public’s focus shifted to epidemic prevention measures in workplaces and schools. During this phase, as the number of infections increased and post-infection symptoms began to appear, topics related to medical treatment and medication saw a significant rise. There was also considerable attention to the government’s efforts in medical and pharmaceutical support. Meanwhile, public concern over economic development continued to grow. After a longer period of adjusted control measures, topics related to the economy, such as employment and consumption, became more prominent. Nonetheless, the public’s demand for medical resources remained strong, and there was a heightened vigilance regarding the mutation of the coronavirus and other infectious diseases. Additionally, there were concerns expressed about the handling of personal data collected during the epidemic.

### 4.2 Comparison with prior work

This study, compared to previous research on the early-stage public opinion related to the implementation of emergency measures for public health crises, found that earlier studies suggested that implementing containment measures in the early stages of an outbreak helped to foster positive public sentiment (29). However, they did not investigate the long-term effects of these measures. In contrast, this study discovered that after prolonged and intensive implementation of containment measures, public sentiment tended to turn negative due to issues encountered in work and daily life. Furthermore, while previous research identified changes in infection rates and the effectiveness of epidemic prevention measures as important factors influencing public sentiment (14), this study highlights that these factors remain crucial, but the impact of control measures on economic development and social life is also a very significant factor.

This study, compared to the research conducted by Samuel (15) on the changes in public opinion regarding the adjustment of pandemic control measures in the United States, finds that China’s top-down adjustment strategy (where policies are issued by the central government and implemented by local governments) and the United States’ bottom-up strategy (where individual states gradually lifted restrictions before the federal government declared the end of lockdowns) have both similarities and significant differences in their impact on public opinion. The similarity lies in the fact that, regardless of the strategy, the public weighs the economic benefits of lifting the lockdown against the health risks. In the long run, supporting the lifting of the lockdown often becomes the mainstream view. However, the differences between the two strategies are also evident. In the U.S., the bottom-up approach lacked unified organization and planning, which often led to the simultaneous emergence of infection, medical resource shortages, and unemployment, creating a compounded negative sentiment. In contrast, China’s top-down approach is more orderly. During the lockdown, the public’s main concern was living conditions, while after the restrictions were eased, the focus shifted to health issues. These two concerns were to some extent separated. Additionally, Samuel only collected data for 9 days following the adjustment of control measures, which merely captured the “momentary excitement” effect of the public in response to the policy changes. In contrast, this study collected data for a total of 156 days before and after the policy adjustments, revealing the public’s pattern of “momentary excitement, short-term negativity, and long-term positivity” in response to the changes in pandemic control measures.

### 4.3 Recommendations

Based on the above research conclusions, the following suggestions are proposed for the governance of public opinion and the further improvement of measures during the adjustment process of response strategies for major public health emergencies.

In the initial phase of adjusting measures for major public health emergencies, responding to public opinion is the most challenging task. At this time, the intensity of public opinion reaches its peak, and public sentiment is at their lowest. To effectively address this, the primary task is to scientifically explain to the public the background and reasons for the adjustment of measures. It is also essential to invite medical experts to disseminate medical knowledge, teach personal protection methods, and demonstrate the ample reserves of medical resources, such as medications, to alleviate excessive public panic. Simultaneously, leveraging the influence of enterprises and well-known public figures online to release positive information about resuming work, production, and economic growth can guide public opinion in a positive direction. Creating a festive atmosphere during major holidays is also an effective way to relieve public tension. Publishing clearer and more specific adjustment plans, based on widely listening to and incorporating public opinion, helps quickly reduce the intensity of public opinion. Additionally, timely responses to specific public concerns, such as the handling of privacy data, are crucial to gaining public trust. For different regions, especially those with lower economic and medical standards and densely populated megacities, more targeted public opinion management measures should be taken. International communication should also be strengthened to avoid misunderstandings about domestic policies due to information delays, which could negatively affect domestic public opinion. Lastly, special attention should be paid to the emotional changes among women during major public health emergencies, providing psychological counseling support when necessary.

Measures for major public health emergencies should be adjusted in a planned and organized manner to avoid a large-scale outbreak of infections within a short period of time, which could lead to strained medical resources. Before adjusting measures, adequate preparations must be made, such as promoting widespread vaccination to enhance population immunity. Sufficient medications and other medical resources should be stockpiled to ensure that infected individuals, especially the older adult and critically ill patients, can receive timely treatment after the measures are adjusted. Lastly, it is crucial to strengthen the protection of collected public privacy data and ensure the legality and security of its use.

### 4.4 Limitations and future work

This research analyzed the characteristics of the evolution of online public opinion before and after the adjustment of China’s epidemic prevention and control measures, based on user behavior data from search engines and social media, using three indicators: public opinion intensity, public sentiment, and discussion topics. The advantages are the large amount of data and high reliability, and it provides a relatively comprehensive exploration of how macro factors (such as the number of infected people, prevention and control policies, etc.) affect public opinion. However, there are also certain limitations. Due to the lack of demographic information in social media data, this study provides limited insight into the differences in attitudes toward the adjustment of epidemic control measures among people of different ages, genders, and levels of health literacy. Future research can combine multiple methods—such as social media text mining, questionnaires, and interviews—to further explore the underlying mechanisms influencing the evolution of online public opinion before and after the adjustment of response measures for major public health emergencies.

## 5 Conclusion

This study employed web data mining methods to thoroughly analyze the evolution characteristics of online public opinion in China before and after the adjustment of COVID-19 pandemic prevention and control measures, focusing on the dimensions of intensity, sentiment, and topics. Based on the research findings, a series of recommendations were proposed for optimizing the response strategies and managing public opinion during the adjustment process of major public health emergency measures.

From an academic perspective, this research compensated for the previous lack of attention from scholars to the changes in public opinion during the process of adjusting measures for responding to public health emergencies from strict to lenient, providing valuable exploration for in-depth research on online public opinion management related to major public health emergencies. From a practical perspective, the conclusions of this study not only offered important references for addressing public opinion management issues and improving related measures when adjusting major public health emergency response strategies in China in the future but also provide valuable experiences that other countries can learn from.

## Data Availability

The original contributions presented in the study are included in the article/supplementary material, further inquiries can be directed to the corresponding authors.
